# Dr. Shute on Medical Science and Practice

**Published:** 1827-10

**Authors:** 


*Principles of Medical Science and Practice.* By HARDWICKE SHUTE, M.D. Physician to the General Infirmary, and to the County and City Lunatic Asylum, Gloucester. Vol. I. Physiology. Octavo, pp. 565. 1824.—Vol. II. Pathology. Octavo, pp. 604. 1826. London and Gloucester.

THE objects of this work are, to use the author's own language, to give a systematic view of the actions of the body in health and disease; to trace these actions to their causes and effects; to ascertain in what manner the healthy action of one organ contributes to the healthy action of another, the diseased action of one organ to the diseased action of another, and the diseased action of one organ to the healthy action of another; to point out the connexion between the natural and artificial cure of diseases, and from this connexion to deduce certain principles, which may impart rationality to our practice, and unite the experience of empiricism with the theory of science.

"The fair, candid, and manly criticism* of a mind anxious only to promote the cause of science, and of medical science in particular, is the great bulwark of our profession. To such criticism I most willingly submit opinions, which, if correct, ought not to be hoarded, and which, if proved to be erroneous, shall be abandoned. Having fully stated my case, and having ascertained the objections which will be made by my learned friend on the other side of the question, I shall avail myself of the privilege of the Bar, and offer some observations in reply; but such reply will be purposely delayed until *the whole of these objections* can be concentrated into a tangible form, and subjected, in their turn, to the ordeal of criticism."—*Preface, p.* v.

We present our readers with the following extract, which is a summary of the author's physiological opinions, and that we may not weaken their effect, we shall abstain from all comment.

"Life is a certain unknown principle superadded to organization, wholly independent of matter for its existence, but altogether dependent upon matter for its union with the body. The only function of life is to preserve the organization of matter. Life and living action are not the same things, the former being independent of matter for its existence, the latter being a property of matter itself; the agency of the former

* Dr. Shute, like all young and unpractised writers, is anxious to multiply epithets. He should have recollected that whatever is fair must be candid; and that every thing which is manly, pre-supposes, as an inevitable consequence, fairness and candour. Can criticism be manly when it is neither fair nor candid?—*Rev.*

being to counteract, and of the latter to promote, a change in the organization of matter. All living action is motion—all living action is dependent upon, and regulated by, the state of the organization. The immediate stimulus to every action is a change in the relative situation of the organic particles. Some organs, as the voluntary muscles, are always in a state of tension, and, therefore, always possess the stimulus to action in themselves. The action itself is a counteracting change in the relative situation of the organic particles, by which the effect of the stimulus is counteracted, and the organic particles contract upon themselves. The power of an organ is that property upon which the counteracting change or re-action is dependent, and corresponds with the nature of the organization. The immediate effect of living action is to accelerate the disorganization of the body, and more particularly the expenditure of the aërial influence; but the support and renewal of the organization are the effects of the combined actions of the system. Thus the action of an individual organ leads to its own disorganization, but at the same time contributes to the support of the organization generally. The action of the stomach, for instance, has a tendency to destroy its own organization, but at the same time produces that change in the food, which renders it capable of being assimilated with the system generally, or with itself individually, through the medium of other organs. The action of the heart, in its immediate effect, has a tendency to destroy its own organization, but at the same time gives an impulse to the blood, by which that fluid is sent to the system generally, and to itself individually, for the purpose of being assimilated with the body through the medium of other organs; and thus the action of an organ contributes, at the same time, to the organization and to the disorganization of its own structure. A constant change in the organization of matter is the effect of this law, which is peculiar to the living system, which defies all imitation, and which has *ignorantly* been attributed to chance. The actions of the body may be arranged according to their *effects*, and reduced to three classes, the locomotive, the digestive, and the sensitive functions. The air may be considered as the food of the body; it must be digested and assimilated with the system, before it can answer the purposes of the animal economy; it is, when digested, the principle upon which living action is more immediately dependent; when united with the blood it constitutes the arterial influence; and when accumulated in the nervous system, is denominated the nervous influence; it is accumulated in the nervous system, for the purpose of contributing to the support of living organization and action, according to the will or to the necessities of the animal; it is the source of animal heat, not in consequence of the change which it undergoes in the lungs, but of the change which it undergoes in every part of the system; it contributes to the support of corporeal and mental action, by supporting the irritability both of the body and of the mind. The nervous system may be regarded as a secondary circulating system, by which the arterial influence is conveyed to the several organs of the body. The ganglia, spinal marrow, and brain, may be considered as so many reservoirs of the arterial influence. The action of nerves and of arteries appears to be the same. Nerves are not the *exclusive* organs of sensation. The brain is the organ of the mind indirectly; it contributes to the support of mental action, in the same manner as nerves contribute to the support of voluntary motion; and, lastly, the action of the nervous system is always from the brain to the extremities of nerves, and *never* from the extremities of nerves to the brain."—*Vol.* I. *p.* 561.

We are now to introduce Dr. Shute as a pathologist. With him we hope "ambition is virtue;" for while men, whose minds will be the light of other ages, are content to be, according to their genius and studies, either physiologists or pathologists, he aspires to be both, and, Cæsar-like, must be every thing, or nothing.*

"We presume, therefore, that all fevers originate in inordinate or excessive atmospherical expenditure—that all fevers depend upon an exhausted state of the brain—that all fevers are attended with inordinate determination of the nervous influence to the capillary extremities—that, in all fevers, the action of the capillary extremities, supported by the inordinate determination of the nervous influence to these organs, is superior to that of the heart—that, in all fevers, the heart is in a state of debility, its increased action being dependent on an increased supply of its stimulus, and regulated by the action of the capillary extremities—that all fevers are attended with an *irregular* distribution of the nervous influence, the increased action of the vascular system (evidently a salutary process) being supported at the expense of other functions, which are not so immediately necessary to life—that the objects of re-action are, in all fevers, increased determination of venous or de-oxygenated blood to the lungs, (for the purpose of acquiring, in greater quantity, that which had been inordinately expended,) and of arterial or oxygenated blood to the brain, the latter process taking place, for the purpose of restoring the brain to its healthy standard of accumulation, and of removing that state of nervous or atmospherical exhaustion, which had been the immediate cause of the febrile reaction—and, finally, that there is reason to suppose, that the duration of all fevers is immediately regulated by the state of the brain, such fevers originating in exhaustion of the brain, and ceasing with the healthy accumulation of the atmospherical principle in that organ."—*Vol.* II. *p.* 328.

---

* The author should ponder on the fate of Boerhaave. "There never was a man, perhaps, more followed and admired in physiology than Boerhaave. I remember the veneration he was held in; and now, in the space of forty years, his physiology is——it shocks me to think in what light it appears."—*Dr. Hunter's Introductory Lecture, p.* 98.

Boerhaave was also thought a great pathologist, yet so fleeting is human distinction, and so true it is that whatsoever is false must inevitably perish, his pathology and physiology lie in a common grave, scarcely known, and, when known, disregarded.—*Rev.*

Yet a little while, and we relinquish the pen; but solicitous, for reasons which will soon be apparent, to do the author justice, even to a Roman strictness, we must indulge in two short extracts.

"We have evidence of inordinate action, and, therefore, of inordinate atmospherical expenditure, in all cases of hectic fever. If the action of a part be excessive, a supply of the atmospherical principle, equal to the expenditure, can only be acquired by the inordinate excitement of *inflammatory* fever; if, however, the expenditure be moderate, but still exceeding the natural and ordinary supply, the action of the heart and arteries (kept up, as in other fevers, by inordinate determination of the nervous influence to the capillary extremities) will be increased in the ratio of the modified difference between the expenditure and the supply. *Hectic*, according to this view of the subject, is a modification of inflammatory fever. *Such* inflammatory fever may continue for months without exhaustion of the system, because the expenditure and the supply are nearly balanced; so nearly balanced, indeed, that, in the natural period of accumulation, I mean in the period of natural repose, the supply not unfrequently exceeds the expenditure. Hence the morning remissions of hectic fever, which, indeed, are often so considerable, as to have induced some writers to denominate hectic 'an irregular intermittent.'"—*Vol.* II. *p.* 330.

"In our observations upon fever we had occasion to remark, that there are three distinct kinds of debility connected with different states of the cerebral organ; a debility of cerebral exhaustion, the power of directing the nervous influence to the support of febrile re-action remaining; a debility of cerebral exhaustion, such power being lost; a debility of cerebral accumulation, the power remaining, but not exerted, because unnecessary. This distinction is the key to our general principles, and by it we shall endeavour to solve the difficulties, and explain the phenomena of constitutional affections, that is to say, of febrile, paralytic, and spasmodic diseases."—*Vol.* II. *p.* 448.

That Dr. Shute is a respectable physician and that he is eminently useful in the station which he fills, we entertain no doubt; and that he is attached to his profession, and possesses considerable ability, we are well convinced from an attentive perusal of his work. We wish we could stop here, but it is impossible without violating truth:—we are the unqualified opponents of his new system of physiology and pathology; we differ with him *toto cœlo;* we consider his opinions to be fanciful, unplausible, unsound, and altogether untenable. In accordance with the sentiments expressed in the preface, Dr. Shute may call on us to refute his numerous and extraordinary opinions; opinions advanced without proof, explained without perspicuity, and advocated in utter defiance of all true logic. Admitting, however, that we were willing to enter the lists, yet, scattered as these opinions are over nearly twelve hundred pages, what just excuse could we offer to our readers for such an employment of time and space? If, indeed, his physiological and pathological opinions were of general currency; if he himself were well known to Fame; if they were exercising a pernicious influence over the minds of men, then would it be a duty, though certainly not a pleasure, to examine them, and in examining, to expose their fallacy. But here circumstances are very different; no duty urges us; no inclinations lead us; and we thank God they do not, since it is, at all times, unpleasant to hold the mirror of critical truth before an author's eyes, to display him there unto himself in all his weakness; to strive to dispel illusions which have been long and fondly cherished, and to attempt to dispossess him of that self-partiality which has been the bane of thousands.

